# Prediction of resistance to hydroxyurea therapy in patients with polycythemia vera: a machine learning study (PV-AIM) validated in a prospective interventional phase IV trial (HU-F-AIM)

**DOI:** 10.1038/s41375-025-02623-5

**Published:** 2025-04-25

**Authors:** Florian H. Heidel, Valerio De Stefano, Matthias Zaiss, Jens Kisro, Eva Gückel, Susanne Großer, Mike W. Zuurman, Kirsi Manz, Kenneth Bryan, Armita Afsharinejad, Martin Griesshammer, Jean-Jacques Kiladjian

**Affiliations:** 1 Hematology, Hemostasis, Oncology and Stem Cell Transplantation, Hannover Medical School (MHH), Hannover, Germany; 2 Leibniz Institute on Aging, Fritz-Lipmann-Institute, Jena, Germany; 3 Sezione di Ematologia, Dipartimento di Scienze Radiologiche ed Ematologiche, Università Cattolica, Fondazione Policlinico A. Gemelli IRCCS, Roma, Italy; 4 Praxis Interdisziplinäre Onkologie und Hämatologie, Freiburg, Germany; 5 Luebecker Onkologische Schwerpunktpraxis, Luebeck, Germany; 6 Novartis Pharma GmbH, Nuremberg, Germany; 7 Novartis Ireland Limited, Dublin, Ireland; 8 University Clinic for Hematology, Oncology, Hemostaseology and Palliative Care, Johannes Wesling Medical Center Minden, UKRUB, University of Bochum, Bochum, Germany; 9 Université de Paris, AP-HP, Hôpital Saint-Louis, Centre d’Investigations Cliniques, INSERM, CIC1427, Paris, France

**Keywords:** Risk factors, Myeloproliferative disease, Clinical trials

## Abstract

Polycythemia vera (PV) is a myeloproliferative neoplasm associated with increased thromboembolic (TE) risk and hematologic complications. Hydroxyurea (HU) serves as the most frequently used first-line cytoreductive therapy worldwide; however, resistance to HU (HU-RES) develops in a significant subset of patients, leading to increased morbidity and necessitating alternative treatments. This study, part of the PV-AIM project, employed machine learning techniques on real-world evidence (RWE) from the Optum® EHR database containing 82.960 PV patients to identify baseline predictors of HU-RES within the first 6–9 months of therapy. Using a Random Forest model, the study analyzed data from 1850 patients, focusing on laboratory parameters and clinical characteristics. Key predictive markers included red cell distribution width (RDW) and hemoglobin (HGB), showing the strongest association with HU-RES. A synergistic interaction between RDW and HGB was identified, enabling TE risk stratification. This study provides a robust framework for early detection of HU-RES using readily available clinical data, facilitating timely intervention. These findings underscore the importance of personalized treatment approaches in managing PV and highlight the utility of machine learning in enhancing predictive accuracy and clinical outcomes. Based on the results of PV-AIM we initiated an open-label, prospective, single-arm, interventional, phase IV study (HU-F-AIM) evaluating HU-resistance/intolerance. Validation of predictive biomarkers may facilitate identification of patients at risk of HU resistance who may benefit from alternative treatment options, possibly preventing ongoing phlebotomy during HU treatment, a frequent therapeutic choice in high-risk PV associated with early disease progression and increased thromboembolic complications. We propose an updated terminology that differentiates between true molecular resistance and clinical resistance, that may indicate the requirement for alternative therapeutic strategies.

## Introduction

Polycythemia vera (PV) is a myeloproliferative neoplasm (MPN) marked by acquisition of an activating mutation of Janus kinase-2 (*JAK2*) [1, 2], which leads to erythrocytosis and frequently leukocytosis and thrombocytosis [3], and is associated with a high symptom burden [4, 5] and disease progression [6]. One of the most significant complications associated with PV is the occurrence of thromboembolic events (TEs) [7]. Risk for TE-complications is increased by specific factors, including age and a history of previous thrombosis [7].

The PV-AIM (Polycythemia Vera Advanced Integrated Models) study utilizes machine learning to analyze real-world evidence, aiming to identify predictive factors for patients with MPN. Recently, the PV-AIM study aimed to identify risk markers in patients with PV undergoing treatment with hydroxyurea (HU) or HU followed by ruxolitinib (RUX) [8]. Using machine learning and real-world evidence from the Optum® EHR database [9], in a first step, the study identified key laboratory markers, including neutrophil-lymphocyte ratio and red cell distribution width (RDW), that correlate with TE risk. The findings support the use of routine laboratory data and decision trees to stratify patient risk, offering a personalized approach to PV management and enhancing timely treatment adjustments to reduce TE incidence. A critical factor in managing the risk of TEs in PV patients is controlling hematocrit (HCT) levels. Uncontrolled HCT levels >45% are closely associated with an increased likelihood of cardiovascular events. Maintaining HCT levels <45% is, therefore, essential to reducing this risk [10, 11]. Common first-line treatments include phlebotomy [12], the use of low-dose aspirin, and/or hydroxyurea (HU) [13, 14]. However, not all patients respond sufficiently to HU and may develop resistance or intolerance to HU [15], rendering the treatment ineffective [16]. HU resistance (HU-RES) increases the risk of disease progression resulting in higher mortality [14, 17, 18]. Therefore, early identification of HU-RES will enable patients to be switched to alternative treatments promptly. One such alternative is RUX, a potent and selective JAK1/2 inhibitor. RUX has shown the ability to provide durable responses and effective hematologic control in PV patients who are resistant to or intolerant to HU [19–21] and reduces the risk for TE complications and progression.

In this analysis of the PV-AIM study, we utilized machine learning techniques to analyze real-world evidence (RWE) with the goal of identifying baseline variables that could predict HU-RES within 6–9 months of starting HU treatment. We identified predictive value for commonly available markers RDW and HGB regarding development of HU-RES. Leveraging these predictive insights will facilitate earlier intervention, optimizing treatment strategies and improving the prognosis for patients with PV who are at risk of HU-RES. We therefore initiated a prospective interventional phase IV trial (HU-F-AIM; NCT05853458) to validate these findings in the real-world clinical setting. This study will evaluate specific HU failure predictors, namely HGB levels of <15.5 g/dL and RDW of >17%, as identified in the PV-AIM analysis, further establishing a robust predictive model for guiding PV management.

## Material and methods

### Machine learning study design PV-AIM

The PV-AIM study is an analytical, descriptive, non-interventional, retrospective cohort analysis of patients with polycythemia vera (PV), utilizing data from the Optum® Electronic Health Records (EHR) database [9]. The Optum® Electronic Health Records (EHR) database is a comprehensive resource that aggregates anonymized patient-level data from a network of healthcare providers across the United States. It includes detailed longitudinal information on demographics, diagnoses, clinical observations, laboratory results, medications, and procedures, allowing researchers to study diverse populations and healthcare outcomes.

The Optum® de-identified EHR dataset provides comprehensive electronic medical records for approximately 105 million patients spanning the years 2007–2020. This dataset captures a representative distribution of patients across the main geographical regions of the United States (West, Midwest, Northeast, and South), with at least ten million patients in each region and proportions of age, gender, ethnicity, and race closely aligning with those of the overall U.S. population. Within the database, records from 82,960 patients diagnosed with PV are included, with a median observation period of 8.4 years. The dataset encompasses detailed information on diagnoses (including PV, other MPNs and complications associated data), demographics, treatments (notably HU and RUX), procedures, laboratory tests, and clinical signs and symptoms. These data are sourced from diverse healthcare settings, including physician offices, emergency departments, laboratories, and hospitals, providing insights into both outpatient and inpatient care.

For this study, data were retrieved from the Optum® EHR database covering the period 2007–2019. This dataset aggregates de-identified clinical and medical administrative records from over 65 healthcare delivery organizations across all 50 U.S. states, encompassing data from more than 150,000 providers, 7000 clinics, and 2000 hospitals. Contributing organizations provide information captured by their local EHR systems. Longitudinal data analysis indicates that the average follow-up duration for patients ranges from at least one year (~66%) to five years or more (~38%), facilitating robust temporal analyses. By integrating data from multiple care settings, this database supports robust epidemiological research, health economics studies, and the evaluation of real-world treatment effectiveness and safety.

The extracted patient data included information on demographics, history of TE events, history of phlebotomy, clinical observations, laboratory results, and use of anticoagulants (Table 1). Since all patient data from the Optum® EHR database were de-identified, Institutional Review Board (IRB) or Ethics Committee approval was not required for this study.

**Table 1:** Baseline characteristics of patient cohorts included in model development and analysis.

	HU-RES (*N* = 733)	NON-RES (*N* = 571)	Overall (*N* = 1304)
Gender, *n* (%)			
Female	307 (41.9)	328 (57.4)	635 (48.7)
Male	426 (58.1)	243 (42.6)	669 (51.3)
Age at index (years), mean (SD)	68.4 (11.2)	71.2 (10.8)	69.6 (11.1)
History of TE, *n* (%)			
Yes	107 (14.6)	93 (16.3)	200 (15.3)
No	626 (85.4)	478 (83.7)	1104 (84.7)
Number of phlebotomies (annualized), mean (SD)	0.74 (1.44)	0.28 (0.77)	0.54 (1.22)
ANC (×10^9^/L), mean (SD)	9.58 (6.00)	8.36 (5.58)	9.05 (5.85)
HCT (%), mean (SD)	49.0 (7.40)	47.2 (7.37)	48.2 (7.43)
HGB (g/dl), mean (SD)	15.6 (2.61)	15.4 (2.49)	15.5 (2.56)
NLR, mean (SD)	6.72 (6.37)	6.06 (5.83)	6.43 (6.15)
RDW (%), mean (SD)	18.1 (3.22)	17.1 (3.05)	177 (3.18)
WBC (×10^9^/L), mean (SD)	13.1 (9.02)	11.1 (6.36)	12.3 (8.03)

### PV-AIM inclusion criteria

The inclusion criteria for the study required that patients be at least 18 years old, have a PV diagnosis and treated exclusively with HU for a duration of 9 months. Additionally, patients needed to have a documented medical history for at least 6 months prior to the index date and a follow-up period of 12 months post-index. Only patients whose first HU treatment occurred after their initial PV diagnosis were considered. However, patients were excluded if they had fewer than two prescriptions for HU or RUX, had been diagnosed with myelofibrosis (MF) or essential thrombocythemia (ET), or had received other cytoreductive treatments such as interferon alpha or busulfan.

### PV-AIM model

A Random Forest ensemble machine learning classification model was developed to predict HU-RES in patients during the 6–9-month post-index period using pre-index patient data. The model’s prediction of HU-RES was based on the European LeukemiaNet (ELN) consensus definition for HU-RES [18], with the ELN criteria adapted to accommodate real-world evidence. To ensure robustness, the model was trained and validated using an 80:20 train-to-validation split, with performance evaluated through 5-fold cross-validation. The model’s accuracy was assessed by the area under the receiver operating characteristic curve (ROC-AUC). Additionally, a feature importance metric was used to identify and rank the ten most influential variables impacting HU-RES prediction. Further analysis involved examining all pairwise combinations of these top ten variables across different thresholds to determine their association with HU-RES. A synergy scoring metric to rank variable in terms of synergy (S): Sab = (Pa * Pb)/Pab where, for a given patient cohort, Pa and Pb are the maximum possible (logrank derived) *p*-values for variable a and variable b and Pab is the maximum *p*-value possible from the combination of variables a and b. Pairs of variables that demonstrated a higher-than-expected synergistic effect in predicting HU-RES were analyzed in more detail, and optimal thresholds were identified for each variable in these pairs. This comprehensive approach aimed to enhance the model’s predictive capability and provide valuable insights into the factors contributing to HU-RES.

### PV-AIM statistical analysis

Data for analysis, including absolute values, binary (yes/no), and median data, were extracted from the Optum® EHR database. In this study, Kaplan–Meier analyses and log-rank tests were utilized specifically to assess and validate the clinical significance of thresholds derived from the random forest model. In contrast, the random forest model itself was employed exclusively for variable selection and to predict HU-RES outcomes. Clearly distinguishing these analytical roles underscores the complementary nature of these statistical approaches. Kaplan–Meier survival curves and log-rank tests were used to compare time-to-event probability distributions, with interactions between variables also evaluated using log-rank tests (significance set at *p* < 0.05 for all analyses). Although random forest models inherently consider variable interactions, we explicitly performed pairwise interaction analyses to identify clinically meaningful thresholds. This approach was chosen to enhance interpretability and provide clinicians with clearly defined thresholds for decision-making. Explicitly defining these interactions ensures practical utility in clinical settings, allowing for precise stratification of patients based on readily measurable clinical parameters.

Statistical analyses were conducted using the ranger package (version 0.13.1; available at https://cran.r-project.org/web/packages/ranger/index.html, accessed 21 June 2023) and R software (version 4.0.2; R Foundation for Statistical Computing, Vienna, Austria, https://www.R-project.org/, accessed 21 June 2023).

### HU-F-AIM study design

HU-F-AIM is an open-label, prospective, single-arm, interventional, phase IV study evaluating HU resistance or intolerance in patients with PV who meet predictive parameters identified in the machine learning–based PV-AIM study [22]. The study consists of three phases: screening period (14 days), treatment period (observation for HU resistance/intolerance, up to 15 months) and follow-up (FU; includes a 30-day safety visit after the last dose, and a 3-month visit for those who show HU resistance or tolerance during the treatment period). Eligible patients will enter the treatment period and will receive de novo HU. To assess HU resistance/intolerance, it is necessary for each patient to reach their personal MTD (dosing regimen detailed in the protocol) or a dose of ≥2 g/day within the first 3 months of HU treatment. Patient eligibility will be determined by the investigator based on the patient’s medical needs, and standard prescribing guidelines will be followed for HU administration. The starting dose should range between 0.5 and 1.5 g/day of HU on day 1 and will be based on the investigator’s discretion. If a patient shows HU resistance/intolerance during the treatment period and requires a switch to second-line therapy or discontinues the treatment for any reason, an end of treatment (EOT) visit will be performed, and the patient will enter the FU period. If no HU resistance/intolerance is observed during the treatment period, then the patient will complete the EOT visit after 15 months, followed by a 30-day safety FU visit (end of study [EOS]). A schedule of assessments is shown Supplementary Table S1.

Monitoring of HU resistance/intolerance is performed every 6 weeks according to the modified ELN criteria [23] (at the earliest after 3 months for patients who started individual maximum tolerated dose (MTD) or ≥2 g/day at baseline, i.e., starting from Visit 7 to EOT; Supplementary Table S1). Study visits are scheduled to occur at screening, day 1, every 2 weeks until week 12 and then every 6 weeks from weeks 18–60, that is, at week 18, week 24, week 30, week 36, week 42, week 48, week 54, week 60 (EOT), 30 days after the last treatment (safety FU) and 3 months after the last treatment (FU visit; only for patients who show HU resistance/intolerance during the treatment period). These short-visit intervals were implemented to identify HU resistance or intolerance, which would allow for a timely switch to the second-line therapy of choice. The decision to set the study duration to 15 months was based on the findings of the PV-AIM project, which established that the minimum period required to observe HU resistance after the index date was 12 months. A single-arm and open-label design was chosen due to the primary endpoint not requiring a comparator arm and HU being the only study treatment for all patients.

### HU-F-AIM inclusion and exclusion criteria

The trial will include patients ≥18 years with a confirmed PV, who have not received any prior pharmacological cytoreductive therapy and who have provided written informed consent. The trial will exclude patients with post-PV MF or accelerated/blast phase MPN (AP/BP-MPN), a contraindication to HU, with a past medical history of rare hereditary galactose intolerance, total lactase deficiency or glucose-galactose malabsorption, or those with active uncontrolled infections and some active malignancies. Full inclusion and exclusion criteria are detailed in Supplementary Table S2.

### HU-F-AIM assessments & endpoints

The endpoints of this study are listed in Supplementary Table S3. The primary endpoint is the proportion of patients with HU resistance/intolerance within 6–9 months after de novo HU treatment initiation in the presence of the PV-AIM HU-RES predictors at the start of HU treatment. Presence of the PV-AIM HU-resistance predictors is defined as having both HGB < 15.5 g/dL (9.62 mmol/L) and RDW ≥ 17%, measured on day 1. Definition of HU-resistance and -intolerance is based on the modified ELN criteria (Supplementary Table S4) [23]. Secondary endpoints include analyses of the proportion of patients with PV who meet the criteria (PV-AIM HU-RES predictors) before the start of HU treatment, the proportion of patients developing HU-RES/intolerance at any time within the treatment period of 15 months in the presence or absence of the PV-AIM HU-RES predictors at the start of HU treatment, and according to the modified ELN criteria [23], criteria used for the PV-AIM project, and the therapies given after confirmation of HU-RES/intolerance. Particularly, for all patients who develop HU-RES/intolerance according to the modified ELN criteria [23] at any time during the treatment period, the following secondary endpoints will be assessed: the proportion of ‘non-switchers’ (i.e. patients remaining on HU despite meeting the HU-RES/intolerance criteria) compared to ‘switchers’, timepoint of therapy switch (after confirmation of HU-RES/intolerance); reasons for therapy switch/non-switch and therapies applied during the FU period. Key exploratory endpoints will include the proportion of patients with PV and HU-RES/intolerance at any time within the treatment period in the presence or absence of HU-RES/intolerance predictors other than HGB and RDW (RBC, HCT, age at index date, APC, ANC, WBC, weight and time between diagnosis and treatment); and change in MPN Symptom Assessment Form Total Symptom Score (MPN-SAF TSS) from baseline to each visit in patients with HU resistance/intolerance compared with patients without HU resistance/intolerance during the treatment period. Safety will be monitored by assessing physical examinations, vital signs, laboratory assessments including hematology, biochemistry and coagulation and by collecting information on adverse events (AEs) (if any) at every visit.

### HU-F-AIM analyses

The analysis of the proportion of PV patients with HU-RES/intolerance, i.e. the primary endpoint will be evaluated by calculating the rate and the respective 95% confidence interval (CI). Where information on resistance predictors is not available on day 1, data from screening or visit 2 (i.e., −1 to −14 days or up to +2 weeks) will be used to analyze the primary endpoint. Where no data are available on HU-RES/intolerance for a specific patient at the EOT, the respective patient will be excluded from the primary analysis. No formal statistical testing will be applied. For patient-reported outcomes, MPN-SAF TSS results will be compared between patients with or without HU-RES/intolerance within the treatment period. Descriptive statistics (e.g., mean, median) will be used to summarize results at each scheduled assessment time point by patient subgroup (with or without HU-RES/intolerance). Additionally, change from baseline for MPN-SAF TSS at the time of each assessment will be summarized. Patients with an evaluable score before the start of HU treatment and at least one additional evaluable score during the treatment period and/or EOT will be included in these analyses. No formal interim analysis is planned for this trial. A full final analysis will be performed after all patients have completed their EOS visit or prematurely terminated.

### Sample size calculation HU-F-AIM

A sample size of 300 patients will allow for a precision in terms of the width of the respective 95% CI of ±0.072 and is based on the following assumptions: (1) Cytoreductive-/HU-naïve patients with PV: Based on German health insurance data (data not shown), approximately 150 hematologists care for approximately 1200 patients with PV per year, of whom 60% are assumed to be HU eligible (this would result in nearly 700 HU-eligible patients in Germany per year; (2) Target population eligible for the analysis of the primary endpoint: Approximately 40% of all HU-naïve patients with PV are estimated to meet the PV-AIM criteria for HU failure at the start of their de novo HU treatment; and (3) approximately 80% of target patients are expected to show HU failure within 3–6 months after starting their de novo HU treatment.

### HU-F-AIM ethics approval and consent to participate

This clinical trial was designed, and will be implemented, executed and reported in accordance with the International Council for Harmonization Tripartite Guidelines for Good Clinical Practices, with applicable local regulations (including European Directive 2001/20/EC or European Clinical Trial Regulation 536/2014 US CFR 21), and with the ethical principles laid down in the Declaration of Helsinki. Written consent will be obtained from all patients.

## Results

### PV-AIM: machine learning analysis of hydroxyurea resistance in real-world data sets of polycythemia vera patients

**Fig. 1:**
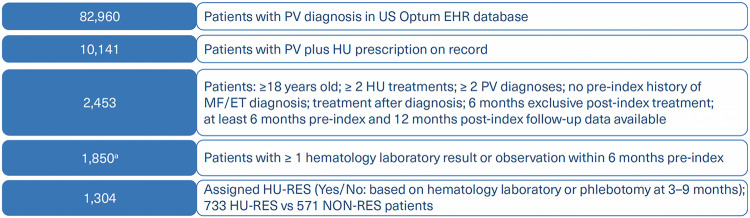
Patient cohorts and analysis. 626 patients with no laboratory values (pre-index 6 months) were not included in this cohort (fewer thromboembolic events (TEs) and phlebotomy than patients included in the final 1304 cohort).

**Fig. 2:**
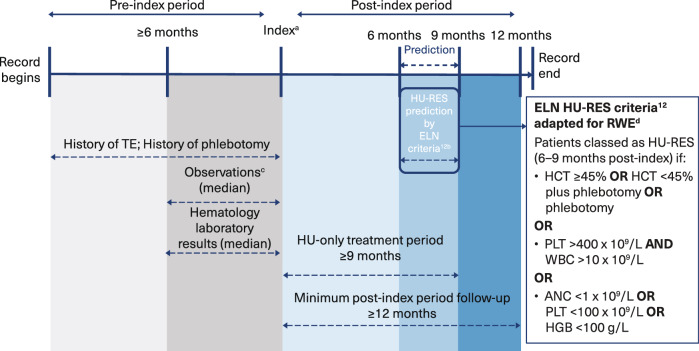
Study design. **a** First hydroxyurea (HU) treatment; **b** Hematocrit, other laboratory assessments, and phlebotomy measured ±14 days in the 6–9 month-post-index window; **c** Observations included: respiratory, heart, pulse, weight, height, body mass index, systolic blood pressure, diastolic blood pressure and demographics (gender, race, ethnicity, region, division, and age at index); **d** Adapted for real world evidence (RWE) with no hydroxyurea dosage applied.

A comprehensive analysis of PV patients was conducted using the Optum® EHR database. Within the database, records from 82,960 patients diagnosed with PV were included. These included 10,141 patients with HU prescription record; 2453 out of those fulfilled the inclusion criteria as outlined above and 1850 had one or more laboratory results or observation within 6 months pre-index (Fig. 1). Out of 1304 that were included in the model development process 733 were included in the HU-RES and 571 in the non-resistant (NON-RES) groups (according to ELN-criteria, Fig. 2, Table 1). This approach not only offers valuable insights into resistance patterns but also supports the application of machine learning tools to predict outcomes and improve patient care in PV management.

**Fig. 3:**
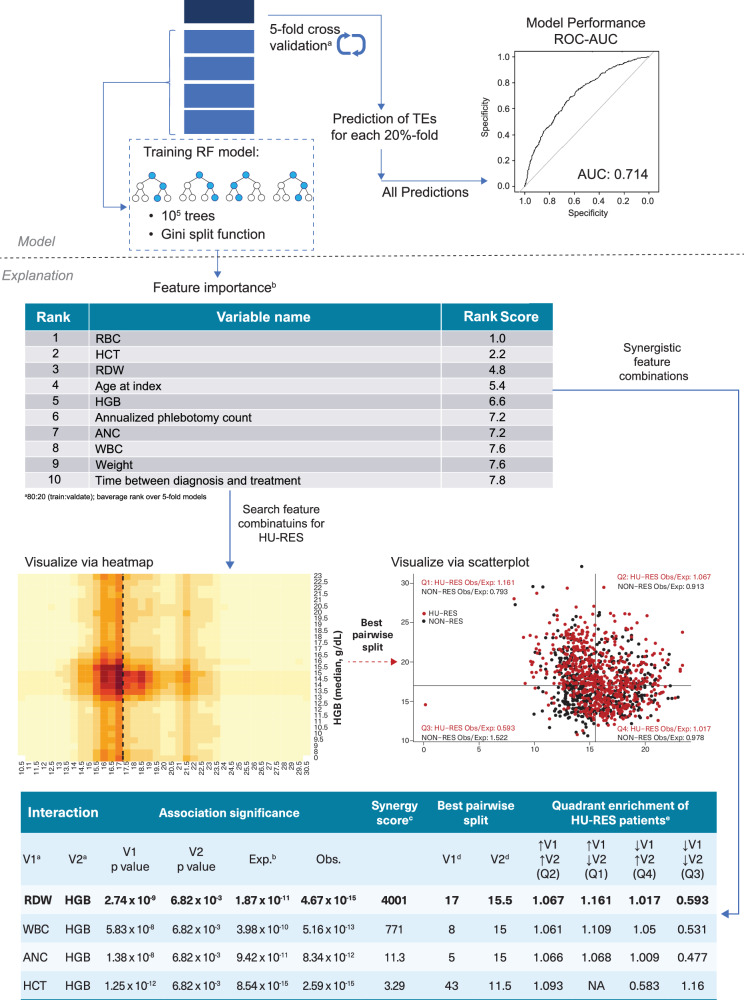
Overview and model explanation. **a** Model development based on 80:20 train:validate; **b** average rank over 5-fold models; ANC absolute neutrophil count, HCT hematocrit, HGB hemoglobin, HU hydroxyurea, HU-RES resistance to HU, NLR neutrophil-lymphocyte ratio, RBC red blood cell count, RDW red cell distribution width, RF random forest, ROC-AUC receiver operating characteristic-area under the curve, TE thromboembolic events, WBC white blood cell count.

The machine learning model developed to predict hydroxyurea resistance (HU-RES) in PV patients demonstrated a composite area under the receiver operating characteristic curve (ROC-AUC) score of 0.71 (Fig. 3). This score reflects a moderate level of accuracy in predicting HU-RES in patients within 6–9 months of starting HU treatment (Fig. 2). While this score indicates that the model is a valuable predictive tool, also suggests improvements can be made to refine the predictive accuracy.

### Analysis of pre-index variables identifies hemoglobin and red cell distribution width as predictors of hydroxyurea-resistance

The model identified ten key pre-index variables that were most predictive of HU-RES (Table 2). These variables were as follows: red blood cell count (RBC), hematocrit (HCT), red cell distribution width (RDW), age at index, hemoglobin (HGB), annualized phlebotomy count, absolute neutrophil count (ANC), WBC, patient weight, and the time between PV diagnosis and initiation of treatment.

**Table 2:** Top ten pre-index variables associated with HU-RES.

Rank	Variable name	Rank score
1	RBC	1.0
2	HCT	2.2
3	RDW	4.8
4	Age at index	5.4
5	HGB	6.6
6	Annualized phlebotomy count	7.2
7	ANC	7.2
8	WBC	7.6
9	Weight	7.6
10	Time between diagnosis and treatment	7.8

Notably, for all of these variables, except “age at index”, higher values were observed in patients who developed resistance to HU compared to those who did not. Interestingly, younger patients (i.e., those with a lower “age at index”) were more likely to develop HU-RES than their older counterparts. This finding highlights that age plays a distinct role in the development of resistance, offering valuable insights into patient risk stratification.

Further analysis revealed that HCT and annualized phlebotomy count had the strongest associations with HU-RES (Table 3). Specifically, a HCT level of ≥44% and an annualized phlebotomy count of ≥0.76 were identified as threshold values most strongly predictive of HU-RES. These values were considered the “best-split” thresholds, providing clinicians with tangible reference points for early identification of patients who are likely to develop HU-RES.

**Table 3:** Significant associations between the top ten pre-index variables.

Variable 1	Variable 2	Association significance (*p*-value)
HCT	Annualized phlebotomy count	1.74 × 10^−22^
RBC	Annualized phlebotomy count	4.26 × 10^−22^
Annualized phlebotomy count	ANC	4.24 × 10^−19^
Annualized phlebotomy count	WBC	5.47 × 10^−19^
HCT	RDW	9.75 × 10^−19^
RBC	Time between diagnosis and treatment	1.59 × 10^−18^
RDW	Annualized phlebotomy count	5.37 × 10^−18^
HCT	Time between diagnosis and treatment	5.60 × 10^−17^
RBC	ANC	6.95 × 10^−17^
Annualized phlebotomy count	Weight	7.17 × 10^−17^

By focusing on these pre-index variables, the machine learning model offers a valuable framework for predicting HU-RES, allowing for earlier intervention and potential adjustments to treatment plans, such as switching to second-line therapies, to mitigate the risks associated with HU-RES in PV patients. Together, the predictive insights generated by this model could significantly improve patient outcomes by preventing disease progression and optimizing long-term treatment effectiveness in patients who do not respond to HU.

In analyzing the interactions between key pre-index variables for predicting HU-RES, the study identified four variable combinations that exhibited significant synergy, meaning their combined predictive power was greater than the sum of their individual effects (Table 4). This synergistic interaction between variables enhances the model’s ability to accurately identify patients at risk of developing HU-RES, which is a crucial step in optimizing treatment for PV. The most notable and impactful of these interactions was between RDW and HGB. This RDW-HGB combination achieved the highest synergy score of 4001, indicating a particularly strong association with HU-RES. The optimal threshold values identified for predicting resistance were an RDW of 17% and an HGB level of 15.5 g/dL, with a highly significant p-value of 4.67 × 10^−15^ (Table 4). This statistical significance underscores the reliability of this combination as a predictor of HU-RES in the patient population.

**Table 4:** Synergistic interactions between variables.

Interaction		Association significance				Synergy Score	Best pairwise split		Quadrant enrichment of HU-RES patients^e^			
V1^a^	V2^a^	V1 *p*-value	V2 *p*-value	Exp.^b^	Obs.		V1^d^	V2^d^	V1⇑ V2⇑ (Q2)	V1⇑ V2⇓ (Q1)	V1⇓ V2⇑ (Q4)	V1⇓ V2⇓ (Q3)
RDW	HGB	2.74 × 10^−9^	6.82 × 10^−3^	1.87 × 10^−11^	4.67 × 10^−15^	4001	17	15.5	1.067	1.161	1.017	0.593
WBC	HGB	5.83 × 10^−8^	6.82 × 10^−3^	3.98 × 10^−10^	5.16 × 10^−13^	771	8	15	1.061	1.109	1.05	0.531
ANC	HGB	1.38 × 10^−8^	6.82 × 10^−3^	9.42 × 10^−11^	8.34 × 10^−12^	11.3	5	15	1.066	1.068	1.009	0.477
HCT	HGB	1.25 × 10^−12^	6.82 × 10^−3^	8.54 × 10^−15^	2.59 × 10^−15^	3.29	43	11.5	1.093	NA	0.583	1.16

^a^Variable median value.

^b^V1 × V2.

^c^Synergy score is expected *p* value/observed *p* value.

^d^Values RDW (%), HGB (g/dL), HCT (%), ANC (×109/L), and WBC (×109/L).

^e^↑variable value above threshold and ↓variable value below threshold (Fig. 4 shows the quadrants for RDW vs HGB).

### Red cell distribution width and hemoglobin allow for clinical risk-stratification of polycythemia vera patients

**Fig. 4:**
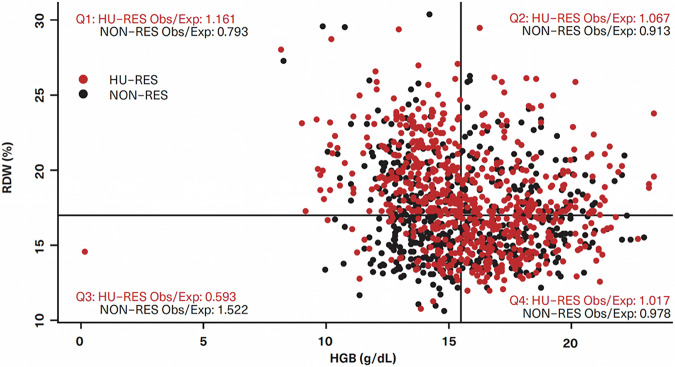
Scatterplot of hydroxyurea resistance (HU-RES) and NON-RES patients according to red cell distribution width (RDW) and hemoglobin (HGB) values. Lines represent the threshold values of 17% for RDW and 15.5 g/dL for HGB dividing the patients into four quadrants (Q).

To better understand and visualize this synergy, patients were categorized into four quadrants based on their RDW and HGB values (Fig. 4). These quadrants allowed for stratification according to the likelihood of developing HU-RES, with those falling within the high-risk thresholds (RDW ≥ 17% and HGB ≤ 15.5 g/dL) showing the greatest susceptibility to resistance. This approach not only highlights the importance of these individual variables but also demonstrates how their interaction can more precisely predict treatment outcomes.

A scatterplot analysis of HU-RES and NON-RES patients according to their RDW and HGB values provides clear insights into the distribution patterns of resistance and non-resistance across different quadrants (Fig. 4). This visualization allows for a more nuanced understanding of how these two variables interact to influence treatment outcomes. Patients were grouped into four quadrants based on their RDW and HGB levels: (i) Quadrant 1 (Q1): RDW ≥ 17% and HGB < 15.5 g/dL. This quadrant represents the highest concentration of patients who developed HU-RES. The combination of elevated RDW (indicative of significant variability in red blood cell size) and lower HGB (reflecting reduced oxygen-carrying capacity) was a strong marker for HU-RES. These patients were shown to be at the highest risk of developing resistance, emphasizing the critical importance of monitoring both RDW and HGB in clinical practice for early detection of potential HU treatment failure. (ii) Quadrant 3 (Q3): RDW < 17% and HGB < 15.5 g/dL. Conversely, the scatterplot revealed that patients with values in this quadrant, where both RDW and HGB levels are lower, represented the highest concentration of NON-RES patients. In this group, the absence of elevated RDW and low HGB indicated a lower likelihood of developing resistance to HU, suggesting that these patients respond more favorably to HU treatment. Overall, this scatterplot analysis underscores the importance of these two biomarkers in the management of PV.

**Fig. 5:**
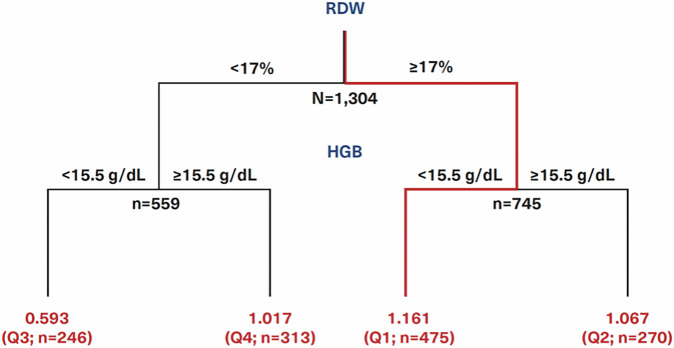
Decision tree. Clinical decision tree based on pre-index red cell distribution width (RDW) and hemoglobin (HGB) values that could help identify patients most likely to become resistant to hydroxyurea (HU) within 6–9 months of starting HU treatment.

The analysis of the relationship between RDW and HGB offers critical insights into the prediction of HU-RES in patients with PV. The significance of the RDW-HGB interaction is depicted across a range of values, highlighting the threshold of 17% RDW as a key point for distinguishing between ‘low RDW’ and ‘high RDW’ populations (Fig. 5). This threshold has a substantial impact on the categorization of patient groups and their respective risk of developing HU-RES. Patients with an RDW of 17% or greater were classified as the ‘high RDW’ group, while those with an RDW < 17% were classified as the ‘low RDW’ group. In particular, the high RDW group was characterized by: (i) Elevated levels of WBC (*p* = 5.66 × 10^−50^), RBC (*p* = 2.84 × 10^−49^), ANC (*p* = 3.99 × 10^−48^), neutrophils (*p* = 2.05 × 10^−25^) and platelets (PLT) (*p* = 2.49 × 10^−9^). These elevated values in the high RDW group suggest an overall increase in cellular activity, which may be indicative of heightened disease activity or a more aggressive disease state, both of which could contribute to HU-RES. (ii) Lower levels of: Lymphocytes (*p* = 5.07 × 10^−36^) and HGB (*p* = 2.72 × 10^−9^). The lower lymphocyte and HGB levels further highlight the distinct hematologic profile of patients with high RDW, which may reflect a compromised immune response and reduced oxygen-carrying capacity. These factors can have a direct impact on disease progression and response.

**Fig. 6:**
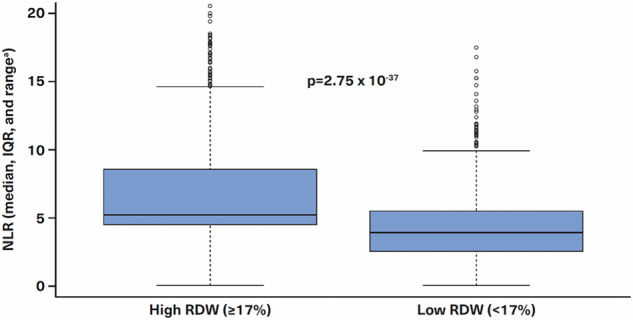
Association with risk factors. Association of neutrophil-lymphocyte-ratio (NLR) with high and low red cell distribution width (RDW) groups.

The proportion of HU-RES patients in the ‘high RDW’ group was found to be significantly higher, 1.36 times greater, than in the ‘low RDW’ group (*p* = 2.74 × 10^−9^), emphasizing the importance of elevated RDW as a marker for predicting treatment resistance. This significant finding supports the use of RDW as a key indicator in identifying patients at higher risk of HU-RES early in their treatment journey. Further analysis of common pre-index laboratory variables between the two groups (high RDW ≥ 17%, *n* = 559; low RDW < 17%, *n* = 745) revealed notable differences, particularly in neutrophil-to-lymphocyte ratio (NLR) (Fig. 6). NLR was significantly elevated in the high RDW group (*p* = 2.75 × 10^−37^), indicating a strong association between elevated RDW and systemic inflammatory markers, as reflected by NLR. This relationship underscores the potential role of inflammation in HU-RES and may point to the underlying biological processes driving the difference in patient outcomes. Additional significant differences between the RDW groups were observed for several other laboratory variables, including WBC, RBC, ANC, PLT, lymphocyte count, and HGB.

### Prospective validation of hydroxyurea-resistance predictors hemoglobin and red cell distribution width in a clinical phase IV trial

**Fig. 7:**
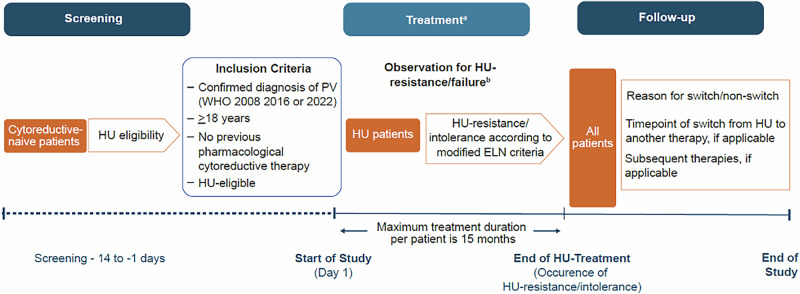
HU-F-AIM study design. ELN European LeukemiaNet, EOS end of study, EOT end of treatment, HU hydroxyurea, PV polycythemia vera, WHO World Health Organization. **a** Visits during the treatment period will be scheduled every 2 weeks for the first 3 months, then every 6 weeks until up to month 15. **b** Assessment of HU-RES/intolerance is based on the modified ELN criteria [23]. If a participant shows HU-RES/intolerance during the treatment period or discontinue the treatment for any reason, an EOT visit will be scheduled, and the participant will enter the follow-up period (i.e. 30-day safety follow-up visit and 3-month follow-up visit [EOS]). If no HU-RES/intolerance is observed during the treatment period of maximum 15 months, then the participant will complete the EOT visit followed by a 30-day safety follow-up visit (EOS).

The results of this machine learning study are currently validated in a prospective Phase IV trial: The HU-F-AIM trial is an open-label, single-arm Phase IV study designed to assess hydroxyurea (HU) resistance or intolerance in PV patients, using predictive parameters identified in prior machine learning research. The study consists of a 14-day screening, up to 15-month treatment with HU, and follow-up for patients showing intolerance or resistance (Fig. 7). Patients receive HU at personalized tolerable doses or ≥2 g/day within 3 months, adjusted biweekly based on blood parameters. Resistance/intolerance is monitored every 6 weeks via modified ELN criteria. Primary and secondary endpoints include rates of HU-RES/intolerance at different time points, effects on symptom scores, and response metrics across subgroups. Safety monitoring and adverse events are tracked at each visit. The sample size of 300 is based on estimated prevalence and HU eligibility among PV patients, aiming to achieve precision with a 95% confidence interval for primary endpoint analysis.

## Discussion

A recent analysis of real-world evidence (PV-AIM study) using machine learning techniques has identified crucial clinical and laboratory variables that can predict HU-RES in patients within the first 6–9 months of starting treatment. Among the key factors influencing HU-RES were pre-treatment HCT levels and the annual frequency of phlebotomies, both of which showed strong associations with the likelihood of developing resistance.

Additionally, a significant synergistic relationship between pre-treatment red cell distribution width (RDW) and hemoglobin (HGB) levels was uncovered. Specific thresholds for these markers, RDW at 17% and HGB at 15.5 g/dL, proved to be highly predictive of HU-RES. The study found that patients with RDW values greater than or equal to 17% and HGB levels below 15.5 g/dL were the most likely to become resistant to HU treatment. Importantly, these variables, RDW and HGB, can be measured easily using standard laboratory tests, making them accessible tools for physicians. By identifying this key combination and its synergistic effect, clinicians can utilize these thresholds as critical markers during routine monitoring of PV patients undergoing HU therapy. Adopting predictive thresholds of RDW ≥ 17% and HGB ≤ 15.5 g/dL could substantially enhance clinical decision-making by identifying patients at risk of developing hydroxyurea resistance earlier in their treatment course. This early stratification would enable clinicians to proactively consider alternative therapeutic strategies, such as transitioning patients to second-line treatments like ruxolitinib, thereby potentially reducing morbidity associated with delayed intervention and improving patient outcomes.

The identified clinical and laboratory parameters, RDW and HGB thresholds, may complement existing molecular markers and other established predictors of hydroxyurea resistance (HU-RES). Molecular mutations, particularly those in DNA repair genes such as TP53 and PPM1D, have been associated with clonal evolution and chemotherapy resistance. These mutations can promote the survival and expansion of therapy-resistant clones, rendering HU treatment less effective over time. Integrating these genetic insights with routinely measured parameters like RDW and HGB offers a more comprehensive risk stratification approach. For instance, while genetic testing can identify patients predisposed to clonal resistance, the identified hematological markers provide dynamic, real-time indicators of emerging resistance during treatment. Additional biomarkers, such as elevated neutrophil-to-lymphocyte ratio (NLR), increased inflammatory cytokines, and aberrant erythropoiesis markers, also play a role in predicting resistance. In particular, elevated HCT levels and frequent phlebotomy requirements reflect ineffective cytoreduction and impending HU-RES, while higher WBC and PLT counts may signify disease progression. Together, these biomarkers highlight both static genetic risks and dynamic hematologic or inflammatory changes. By combining molecular diagnostics with accessible laboratory markers, clinicians can create multi-dimensional predictive models, enhancing early detection and enabling tailored interventions that mitigate the risks of HU-RES and improve outcomes in PV patients.

The retrospective nature of this analysis and the use of real-world data can present some limitations to this analysis. Although data for a large number of patients with PV was available within the Optum^®^ EHR database, strict inclusion and exclusion criteria were applied to obtain a focused cohort of patients for this analysis. Therefore, this analysis population may have excluded some patients of interest that may have predicted HU-RES, such as those who stopped treatment prior to 9 months due to HU-RES. Collection of clinical data for the Optum^®^ EHR database takes place from a wide range of sources, there could be a possibility of missing, invalid or unrecorded data, inaccuracies, and/or technical errors. Recording input codes were also determined using subjective medical judgement. Despite these potential limitations, the outcomes from this analysis will be validated externally through the prospective, single-arm, open-label HU-F-AIM study.

The analysis underscores the value of patient history as an essential resource for clinicians. By proactively scheduling laboratory tests before initiating HU treatment, physicians can better identify patients at higher risk of developing HU-RES. Early identification of at-risk individuals allows for closer monitoring throughout treatment. This, in turn, enables timely and informed clinical decisions, including the potential for an earlier switch to second-line therapies like RUX, should HU-RES develop. Proactive management of these patients can help optimize treatment outcomes and mitigate the risks associated with HU-RES. Given that HU is the most widely used cytoreductive therapy worldwide, these findings have broad clinical relevance. In many regions, including some European countries and non-EU nations, alternative first-line options such as interferons are either not available or not approved, making HU the primary treatment choice. Consequently, the risk scores established in this study, based on widely accessible laboratory standard parameters, will be applicable and useful on a broad scale.

In this study, we propose a refined perspective on the concept of HU-RES in PV. Traditionally, HU-RES has been attributed to intrinsic molecular or immunological mechanisms that directly limit the drug’s efficacy. However, we argue that in PV, resistance should be considered in the context of the disease itself rather than solely as a failure of the molecular inhibition of DNA synthesis. The currently used term “HU resistance” implies a mechanistic failure at the cellular or molecular level, whereas in PV, the persistence of elevated HCT and ongoing need for phlebotomy despite HU therapy may primarily reflect the progressive nature of the disease rather than a molecularly driven loss of drug efficacy. Given this distinction, we propose an updated terminology that differentiates between true molecular resistance, where intrinsic biological mechanisms directly impair HU function, and clinical resistance, where the disease’s trajectory necessitates alternative therapeutic strategies. This nuanced approach aligns better with the heterogeneous response patterns observed in PV and provides a clearer rationale for treatment decisions.

## Supplementary information

Supplemental Data

## Data Availability

Novartis is committed to sharing with qualified external researchers access to patient-level data and supporting clinical documents from eligible studies. These requests are reviewed and approved by an independent review panel on the basis of scientific merit. All data provided are anonymized to respect the privacy of patients who have participated in the trial, in line with applicable laws and regulations. This trial data availability is in accordance with the criteria and process described on www.clinicalstudydatarequest.com.
