# Supplementary material for: Prediction of resistance to hydroxyurea therapy in patients with polycythemia vera: a machine learning study (PV-AIM) validated in a prospective interventional phase IV trial (HU-F-AIM)
Source: Leukemia. 2025 Apr 25;39(7):1692–701. doi: 10.1038/s41375-025-02623-5 (PMC12208875; doi:10.1038/s41375-025-02623-5)
Supplement: Supplementary file 1 — Supplemental Data [file 41375_2025_2623_MOESM1_ESM.docx]

**Supplementary Figures and Tables**

**Prediction of Resistance to Hydroxyurea Therapy in Patients With Polycythemia Vera: A Machine Learning Study (PV-AIM) followed by validation in a prospective interventional phase IV trial (HU-F-AIM)**

Florian H Heidel^1,2^, Valerio De Stefano^3^, Matthias Zaiss,^4^ Jens Kisro,^5^ Eva Gückel,^6^ Susanne Großer,^6^ Mike W Zuurman^5^, Kirsi Manz^1^, Kenneth Bryan^7^, Armita Afsharinejad^7^, Martin Griesshammer^8^, Jean-Jacques Kiladjian^9^

1 Hematology, Hemostasis, Oncology and Stem Cell Transplantation, Hannover Medical School (MHH), Hannover, Germany

2 Leibniz Institute on Aging, Fritz-Lipmann-Institute, Jena, Germany

3 Sezione di Ematologia, Dipartimento di Scienze Radiologiche ed Ematologiche, Università Cattolica, Fondazione Policlinico A. Gemelli IRCCS, Roma, Italy

4 Praxis Interdisziplinäre Onkologie und Hämatologie, Freiburg, Germany

5 Luebecker Onkologische Schwerpunktpraxis, Luebeck, Germany

6 Novartis Pharma AG, Basel, Switzerland

7 Novartis Ireland Limited, Dublin, Ireland

8 University Clinic for Hematology, Oncology, Hemostaseology and Palliative Care, Johannes Wesling Medical Center Minden, UKRUB, University of Bochum, Bochum, Germany

9 Université de Paris, AP-HP, Hôpital Saint-Louis, Centre d’Investigations Cliniques, INSERM, CIC1427, Paris, France

**Table S1.** Schedule of assessments

|  | **Screening** | **Treatment period (±3 days)** | | | **End of treatment visit** | **Follow-up period**  **(±7 days)** | |
| --- | --- | --- | --- | --- | --- | --- | --- |
| **Visit number** |  | **1** | **2‑7** | **8‑14** |  | **30‑day safety follow‑up** | **3‑month follow‑up** |
| **Days/Weeks** | **Days −14 to −1** | **Day 1** | **Every 2 weeks** | **Every 6 weeks** |  |  |  |
| Informed consent | x |  |  |  |  |  |  |
| Inclusion/exclusion criteria | x |  |  |  |  |  |  |
| Patient history |  | | | | | | |
| Demography | x |  |  |  |  |  |  |
| Relevant medical history | x |  |  |  |  |  |  |
| PV diagnosis/disease and treatment history | x |  |  |  |  |  |  |
| Smoking history | x |  |  |  |  |  |  |
| Prior/concomitant medications | x | x | x | x | x | x | x |
| Physical examination | x | x |  | x | x |  |  |
| ECOG performance status | x | x |  | x | x |  |  |
| Height | x |  |  |  |  |  |  |
| Weight/BMI | x |  |  |  |  |  |  |
| Vital signs | x | x | x | x | x |  |  |
| Spleen size | x | x | x | x | x |  |  |
| Biochemistry | x | x | x | x | x |  |  |
| Hematology and coagulation | x | x | x | x | x |  |  |
| Liver or renal follow-up testing* | x |  | x | x | x |  |  |
| hsCRP | x | x | x |  | x |  |  |
| Iron test | x | x | At visit 4  (week 6) and 7 (week 12) | x | x |  |  |
| Thromboembolic events and cardiovascular events | x | x | x | x | x | x | x |
| MPN symptoms by MPN‑10 |  | x | x  At visit 7 (week 12) | x  At visit 9  (week 24) | x |  |  |
| Disease progression | x |  | x | x | x |  |  |
| Pregnancy test (if applicable) | x | x | x | x | x |  |  |
| Treatment |  | | | | | | |
| HU eligibility | x |  |  |  |  |  |  |
| Phlebotomies | x | x |  | x | x |  |  |
| Anti-coagulation | x | x | x | x | x |  |  |
| Study drug administration |  | x | x | x |  |  |  |
| Efficacy assessment |  |  |  |  |  |  |  |
| PV-AIM HU resistance predictors | x | x |  | x | x |  |  |
| HU resistance/intolerance |  |  | x  At visit 7  (week 12) | x | x |  |  |
| Adverse events | x | x | x | x | x | x | x |
| Optional research |  | | | | | | |
| Blood sampling for additional exploratory research |  | x  (before the start of HU) |  |  | x |  |  |
| Drug compliance |  | x | x | x | x |  |  |

*Only for patients with hepatic or renal impairment.

BMI, body mass index; ECOG, Eastern Cooperative Oncology Group; hsCRP, high-sensitivity C-reactive protein; HU, hydroxyurea; MPN, myeloproliferative neoplasm; PV, polycythemia vera.

**Table S2.** Key inclusion and exclusion criteria

| **Key inclusion criteria** |
| --- |
| Signed informed consent must be obtained before participation in the study |
| Patients aged ≥18 years |
| Confirmed diagnosis of PV (according to WHO 2008, 2016 or 2022 criteria) (1-3) |
| ECOG PS ≤2 |
| No previous pharmacologic cytoreductive therapy (including investigational drugs) |
| No phlebotomy in last 14 days |
| HU-eligible   - High-risk: age ≥60 years and/or prior history of thrombosis - Low-risk: showing at least one of the defined criteria:   - Signs of disease progression (myeloproliferation):   - Increase in spleen size or symptomatic splenomegaly  - Platelet increase to >1,000,000/μL  - WBC increase to ≥15,000/μL  - Frequent (>10 per year) or increasing frequency of phlebotomies   - - Increasing risk of thromboembolism and bleeding:   - New thromboembolism and/or hemorrhagic complications  - Microcirculation disorders despite acetyl salicylic acid 2 × 100 mg/day  - Restricted feasibility or intolerance of phlebotomies  - Symptomatic iron deficiency  - Uncontrolled increase in hematocrit  - Severe or distressing disease-related symptoms |
| Female participants of childbearing potential should have a negative serum pregnancy test  within 72 hours prior to receiving the first dose of study treatment |
| **Key exclusion criteria** |
| Patients with post-PV MF or AP/BP-MPN AML |
| Patients with a contraindication to HU according to the Summary of Product Characteristics (severe bone marrow depression, leukopenia (<2.5 × 10^9^ leukocytes/L), thrombocytopenia (<100 × 10^9^ platelets/L), severe anemia (<10 g/dL HGB) |
| Patients with rare hereditary galactose intolerance, total lactase deficiency or glucose-galactose malabsorption in their past medical history |
| Active uncontrolled infection that is considered by the investigator as a reason for exclusion |
| Active malignancies (except for carcinoma in situ; prostate cancer and breast cancer in remission and, where necessary, or ongoing hormonal therapy) |
| Inadequate renal function as demonstrated by Modification of Diet in Renal Disease estimated glomerular filtration rate (MDRDeGFR) <30 mL/min/1.73 m^2^ or on dialysis |
| Pregnant or lactating women, where pregnancy is defined as the state of a female after conception and until the termination of gestation, confirmed by a positive human chorionic gonadotrophin laboratory test |
| Sexually active males unwilling to use a condom during intercourse while participating in the study treatment and for at least 6 months after stopping the study treatment |
| Patients with HIV treated with nucleoside reverse transcriptase inhibitors, including didanosine and stavudine |

AP/BP-MPN AML, accelerated phase/blast phase myeloproliferative neoplasm acute myeloid leukemia; ECOG PS, Eastern Cooperative Oncology Group Performance Status; HBG, hemoglobin; HIV, human immunodeficiency virus; HU, hydroxyurea; MDRDeGFR, Modification of Diet in Renal Disease estimated glomerular filtration rate; PV MF, polycythemia vera myelofibrosis; WBC, white blood cell; WHO, World Health Organization.

**Table S3.** Study endpoints

| **Primary endpoint** |
| --- |
| Proportion of patients with PV and HU resistance/intolerance within 6-9 months after the start of de novo HU treatment in the presence of PV-AIM HU resistance predictors at the start of HU treatment. |
| **Secondary endpoints** |
| Proportion of patients with PV who meet the PV-AIM HU resistance predictors criteria before the start of HU treatment. |
| Proportion of patients developing HU resistance/intolerance at any time within the maximum treatment period of 15 months. |
| Proportion of patients developing HU resistance/intolerance at any time within the maximum treatment period of 15 months in the presence or absence of the PV-AIM HU resistance predictors at the start of HU treatment. |
| For all patients who develop HU resistance/intolerance according to the modified ELN criteria at any time during the maximum treatment period of 15 months.   - Proportion of ‘non-switchers’ (i.e. patients remaining on HU despite they meet the HU resistance/intolerance criteria) compared with that of ‘switchers’. - Timepoint of therapy switch (after confirmation of HU resistance/intolerance). - Reasons for therapy switch/non-switch. - Therapies applied during the follow-up period. |
| **Exploratory endpoints** |
| Proportion of patients with PV and HU resistance/intolerance at any time within the maximum treatment period of 15 months in the presence or absence of HU resistance/intolerance predictors other than hemoglobin and red blood cell distribution width. |
| Change in myeloproliferative neoplasm symptom assessment form total symptom score from baseline to each visit in patients with HU resistance/intolerance compared with patients without HU resistance/intolerance during the maximum treatment period of 15 months. |

PV-AIM HU-resistance predictors: hemoglobin <15.5 g/dL (9.62 mmol/L) and red blood cell distribution width ≥17%. Definition of HU-resistance and intolerance is based on the modified ELN criteria (4).

ELN, European LeukemiaNet; HU, hydroxyurea; PV, polycythemia vera.

**Table S4.** Modified ELN criteria (4)

| **Criteria** | **Protocol** |
| --- | --- |
| 1 | Need for phlebotomy to maintain the hematocrit level <45% after 3 months at a maximum tolerated dose or a dose of ≥2 g/day HU, *or* |
| 2 | Uncontrolled myeloproliferation (i.e. platelet count >400 × 10^9^/L, and white blood cell count >10 × 10^9^/L) after 3 months at a maximum tolerated dose or at a HU dose of ≥2 g/day, *or* |
| 3 | Failure to reduce massive splenomegaly by >50% as measured by palpation or failure to completely relieve symptoms related to splenomegaly after 3 months at a maximum tolerated dose or a dose of ≥2 g/day HU |
| 4 | At the lowest dose of HU required to achieve a complete or partial clinico-hematologic response, any one of: (i) Absolute neutrophil count <1.0 × 10^9^/L; (ii) Platelet count <100 × 10^9^/L; (iii) hemoglobin <10 g/dL |
| 5 | At any HU dose, presence of leg ulcers or other unacceptable HU-related non-hematological toxicities (e.g. mucocutaneous manifestations, gastrointestinal symptoms, pneumonitis or fever) |

AE, adverse event; ELN, European LeukaemiaNet; HU, hydroxyurea.

**References**

1. Tefferi A, Vardiman JW. Classification and diagnosis of myeloproliferative neoplasms: the 2008 World Health Organization criteria and point-of-care diagnostic algorithms. Leukemia. 2008;22(1):14-22.

2. Arber DA, Orazi A, Hasserjian R, Thiele J, Borowitz MJ, Le Beau MM, et al. The 2016 revision to the World Health Organization classification of myeloid neoplasms and acute leukemia. Blood. 2016;127(20):2391-405.

3. Khoury JD, Solary E, Abla O, Akkari Y, Alaggio R, Apperley JF, et al. The 5th edition of the World Health Organization Classification of Haematolymphoid Tumours: Myeloid and Histiocytic/Dendritic Neoplasms. Leukemia. 2022;36(7):1703-19.

4. Passamonti F, Griesshammer M, Palandri F, Egyed M, Benevolo G, Devos T, et al. Ruxolitinib for the treatment of inadequately controlled polycythaemia vera without splenomegaly (RESPONSE-2): a randomised, open-label, phase 3b study. Lancet Oncol. 2017;18(1):88-99.
